# Highly Sensitive FPW-Based Microsystem for Rapid Detection of Tetrahydrocannabinol in Human Urine

**DOI:** 10.3390/s17122760

**Published:** 2017-11-29

**Authors:** Je-Wei Lan, Chia-Hsu Hsieh, I-Yu Huang, Yu-Cheng Lin, Tsung-Yi Tsai, Chua-Chin Wang

**Affiliations:** 1 Department of Electrical Engineering, National Sun Yat-sen University, Kaohsiung 80424, Taiwan; d993010003@student.nsysu.edu.tw (J.-W.L.); tim06061983@gmail.com (C.-H.H.); iyuhuang@mail.nsysu.edu.tw (I.-Y.H.); zack@vlsi.ee.nsysu.edu.tw (T.-Y.T.); 2 Department of Engineering Science, National Cheng Kung University, Tainan 70101, Taiwan; yuclin@mail.ncku.edu.tw

**Keywords:** flexural plate-wave, tetrahydrocannabinol, circular-type interdigital transducer, reflective grating structure, low insertion loss, field-programmable gate array, readout system

## Abstract

This paper presents a highly sensitive flexural plate-wave (FPW)-based microsystem for rapid detection of tetrahydrocannabinol (THC) in human urine. First, a circular-type interdigital transducer (IDT) was integrated with a circular-type silicon-grooved reflective grating structure (RGS) to reduce insertion loss. Then, with lower insertion loss (−38.758 dB), the FPW device was used to develop a novel THC biosensor, and the results reveal that this FPW-THC biosensor has low detection limit (1.5625 ng/mL) and high mass-sensitivity (126.67 cm^2^/g). Finally, this biosensor was integrated with field-programmable gate array (FPGA) board and discrete components for prototyping a FPW readout system, whose maximum error was 12.378 kHz to ensure that the linearity of detection up to R-square is equal to 0.9992.

## 1. Introduction

Drug abuse and addiction have been serious health and social problems in recent years, which also lead to a high crime rate [1,2]. Among all illegal drugs in Taiwan, marijuana is the most controversial one, which affects human both mentally and physically [3]. For example, smoking marijuana will cause increased heartbeat, lowered blood pressure, blunted short-term memories, and lost focus. Therefore, a reliable, effective, and portable screening microsystem used in early detection will certainly reduce, or even prevent, drug abuse and addiction. Tetrahydrocannabinol (THC) is the main ingredient of marijuana, an active chemical in cannabis, and one of the oldest hallucinogenic drugs ever known. Notably, detecting THC concentration is important as well as carcinoembryonic antigen (CEA) concentration for diseases or tumors related to lung, liver, stomach, colorectal, and breast, etc.

Urinanalysis is the main drug testing method among which enzyme-linked immunosorbent assay (ELISA) techniques [4], surface plasmon resonance (SPR) [5], high performance liquid chromatography (HPLC) [6,7] and gas chromatography-mass spectrometry (GC-MS) [8,9,10] are mostly used. Although these four technologies present high sensitivity and accuracy, their expensive instruments, large dimensions, time-consuming sample pretreatment, and lack of real-time monitoring functions limit their applications. To solve the above issues, acoustic sensors have been developed for molecular mass detection in the last two decades [11,12,13,14,15], including thickness shear mode (TSM), surface acoustic wave (SAW), shear horizontal acoustic plate mode (SH-APM), and flexural plate-wave (FPW). Table 1 qualitatively summarizes the characteristics of the four sensor families discussed. For biosensing application in contact with liquid, there are some points to be concerned: (1) high mass sensitivity; (2) particle motions are transverse only, or have phase velocities lower than the speed of sound in the liquid to avoid the energy dissipation; and (3) low operating frequency for easy detecting circuit design. Among the three points concerned above, the FPW sensor is the most suitable for biosensing applications since it has high mass sensitivity at low operating frequency Thus, it is used as the biosensor in this paper.

This paper presents a FPW-based biosensor for rapid detection of THC antigen in urine by using micro-electromechanical systems (MEMS) and cystamine-glutaraldehyde-based self-assembled monolayers (SAMs) technologies. To further justify the detection architecture, this biosensor needs a readout system. Wang et al. reported a high-precision readout system based on FPW sensors, where the resonant frequency shift is proportional to CEA concentration [16]. However, the input range of operational amplifier (OPA) requires at least 20 MHz bandwidth, and using too many OPAs result in higher power consumption and larger layout area. To resolve the bandwidth problem, we used an amplitude to voltage converter (AVC), and since the output DC voltage of AVC is proportional to the amplitude of the input signal, the filter of the previous design composed of OPAs is no longer required.

## 2. Fabrication of FPW-Based THC Biosensor and Design of Readout System Circuit 

### 2.1. Fabrication of FPW-Based THC Biosensor 

**Figure 1:**
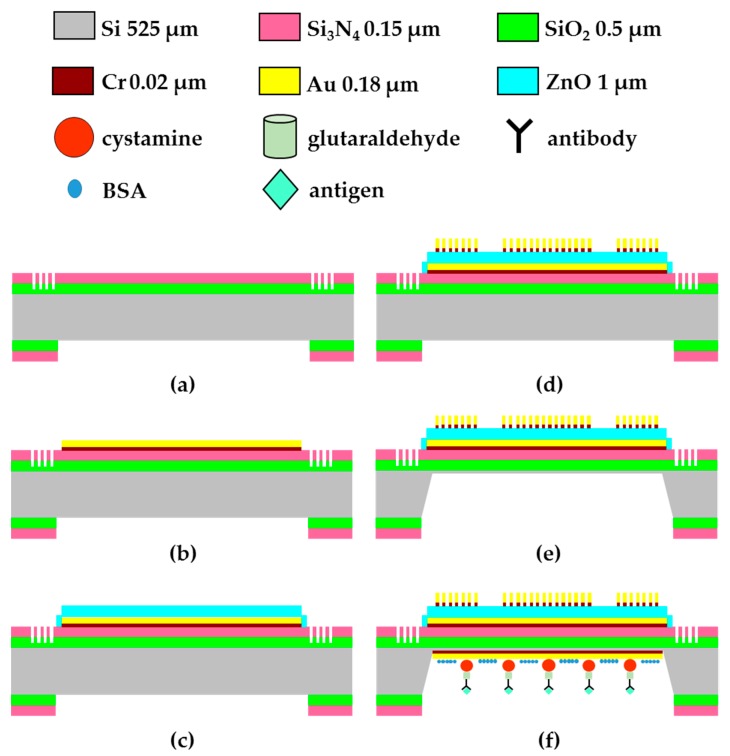
Main processing steps of the proposed FPW device: (**a**) deposit SiO_2_/Si_3_N_4_, etch Si-groove RGS, and pattern backside SiO_2_/Si_3_N_4_; (**b**) deposit and pattern Cr/Au ground electrode; (**c**) deposit and pattern ZnO; (**d**) deposit and pattern Cr/Au IDTs; (**e**) etch the backside silicon by using 30 wt % KOH at 60 °C and 30 wt % KOH at 27 °C; (**f**) the THC antibody has to be coated on the back-side silicon cavity to catch the THC antigen in urine.

The main processing steps of the FPW transducer are shown in Figure 1: (a) deposit SiO_2_/Si_3_N_4_ (0.5/0.15 μm), etch Si-groove RGS (0.3 μm), and pattern backside SiO_2_/Si_3_N_4_; (b) deposit and pattern Cr/Au (0.02/0.15 μm) ground electrode; (c) deposit and pattern ZnO (1 μm); (d) deposit and pattern Cr/Au (0.02/0.18 μm) IDTs; (e) etch the backside silicon by using 30 wt % KOH at 60 °C and 30 wt % KOH at 27 °C; (f) the THC antibody has to be coated on the back-side silicon cavity to catch the THC antigen in urine. For the detailed manufacturing process flows, please refer to [17].

**Figure 2:**
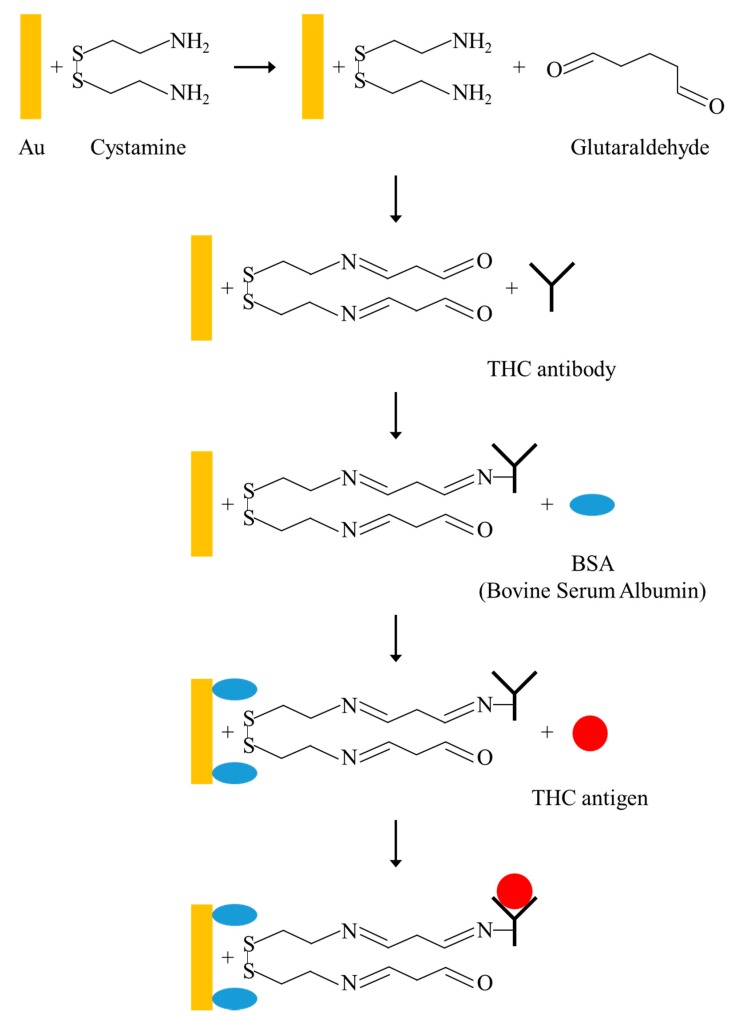
Schematic diagram for the integration of cystamine SAM, glutaraldehyde, THC antibody, and THC antigen in multilayers.

Figure 2 presents the final configurations of the FPW-based biosensor and the integrated cystamine SAM/glutaraldehyde/THC antibody/THC antigen multilayer. SAMs technology is used in the gold (Au) surface with cysteine dialdehyde method (cystamine-glutaraldehyde method) for chemical adsorption, since SAMs have high stability, simple fabrication, and variability of terminal functional groups, and are widely used for the ideal surface of basic scientific research and biological surface science. Cystamine is a solution of molecular compounds with a sulphurated group at one end and with an amine group, NH_2_, at the other end. The sulphurated group bonds to the Au electrode surface to form a covalent bond [18,19,20]. Glutaraldehyde is an organic compound with aldehyde (−CHO) at the both ends, and its main function is to bridge between cystamine and protein antibodies. 

The experimental procedure of the cystamine-glutaraldehyde method is as follows. (1) Immerse the wafer in 20 mM cystamine solution for 1 h and clean with DI water. (2) Immerse the wafer in glutaraldehyde solution for 1 h and clean with DI water. (3) Titrate 10 μL of THC antibody solution onto the upper surface of the Au electrode, which is then exposed to a temperature of 27 °C and a relative humidity of 100% RH for 1 h. The wafer was then cleaned with wash buffer, PBS, and DI water. (4) Titrated 20 μL of BSA solution on the upper surface of the Au electrode, and after a 30-min reaction, the surface is cleaned with wash buffer, PBS, and DI water. (5) Titrated 10 μL of THC urine specimens at six different concentrations (1.5625, 3.125, 7.25, 12.5, 25, and 50 ng/mL) on the upper surface of the Au electrode for measurement.

### 2.2. FPW Readout System Circuit Design

**Figure 3:**
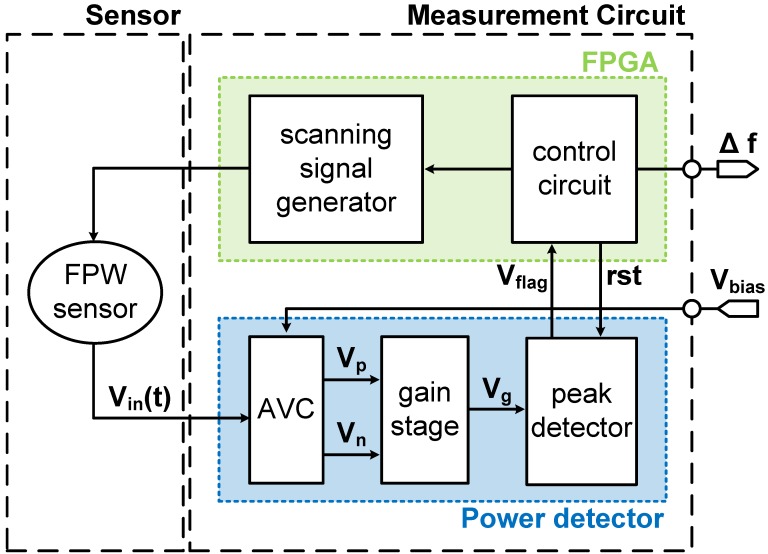
Block diagram of the FPW readout system.

The proposed FPW readout system, as shown in Figure 3, is mainly divided into two parts: the sensor and the measurement circuit. The measurement circuit was composed of a scanning signal generator, and control circuit (FPGA), AVC, gain stage, and peak detector (power detector). Note that the scanning signal generator and control circuit were implemented by FPGA to ensure reliability. The scanning signal generator generated sinusoidal waves with various frequency, which was used as the testing resonance frequency. Featured with that of the input signal and with the resonance frequency, the FPW sensor generated a corresponding output with the highest amplitude. The amplitude to voltage converter (AVC) then transformed AC signals into DC voltages, V_p_ and V_n_. The DC voltages were enlarged in gain stage, and a peak detector [21,22,23,24,25] monitored the output DC voltage V_g_, where V_flag_ was generated to control circuit when the maximum was detected. Note that all the mentioned procedures were calibrated in test cycles. When the FPW sensor detected different concentrations of marijuana, the corresponding V_in_(t) with different frequencies was also detected in the following test cycles. Finally, the control circuit calculated the different resonant frequencies through two test cycles.

**Figure 4:**
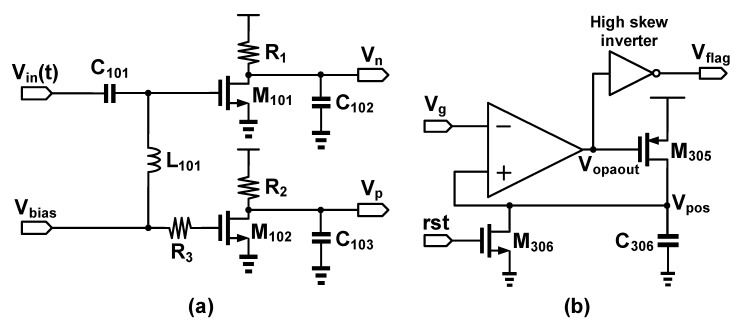
Schematic of (**a**) amplitude-to-voltage converter (AVC) and (**b**) peak detector.

The schematic of AVC, as shown in Figure 4a, has a large capacitor C_101_ filtering out the DC component of V_in_(t). V_bias_ is used to bias M_101_ and M_102_ into saturation region, since V_in_(t) is a relatively small signal. L_101_ is an inductor to isolate AC signal ripples coupled from the upper circuit. Note that the combination of R_1_ and C_102_, and R_2_ and C_103_ act as a low pass filter for DC voltages, V_n_ and V_p_, respectively, where V_p_ is a constant voltage level and V_n_ is a voltage level corresponding to input signal amplitude by contrast. Gain stage comprises two circuits, namely, OPA-based subtractor and OPA-based amplifier. The subtractor generates the difference, V_g_, between V_n_ and V_p_, while the amplifier enlarges V_g_ which is then coupled to the peak detector.

Figure 4b presents the schematic of the peak detector composed of an OPA, a high skew inverter, a capacitor, and two transistors. When V_g_ is larger than V_pos_, V_opaout_ turns on M_305_ to charge C_306_. However, charging C_306_ raises the voltage level of V_pos_ and leads to a negative feedback mechanism. Once if V_g_ is smaller than V_pos_, V_opaout_ is pulled up toward VDD to pull down V_flag_. Therefore, a new peak voltage is detected. Note that M_306_ is used to reset the voltage level of C_306_ at the beginning of any detection.

### 2.3. Preparation of the THC Urine Specimens

This study uses THC urine specimen which was prepared by the following procedures. First, we put 100 μL of THC stock solution (at 100 ng/mL concentration) into a 1.7 mL microcentrifuge tube, and then added 100 μL of negative urine solution into the tube. Finally, we mixed the urine and the THC solution well to prepare 50 ng/mL THC urine specimens for experiment. We also used the serial dilution to prepare THC urine specimens of 25, 12.5, 7.25, 3.125, and 1.5625 ng/mL concentrations.

## 3. Experimental Results and Analysis

### 3.1. Characterization of the Proposed FPW-Based THC Biosensor

**Figure 5:**
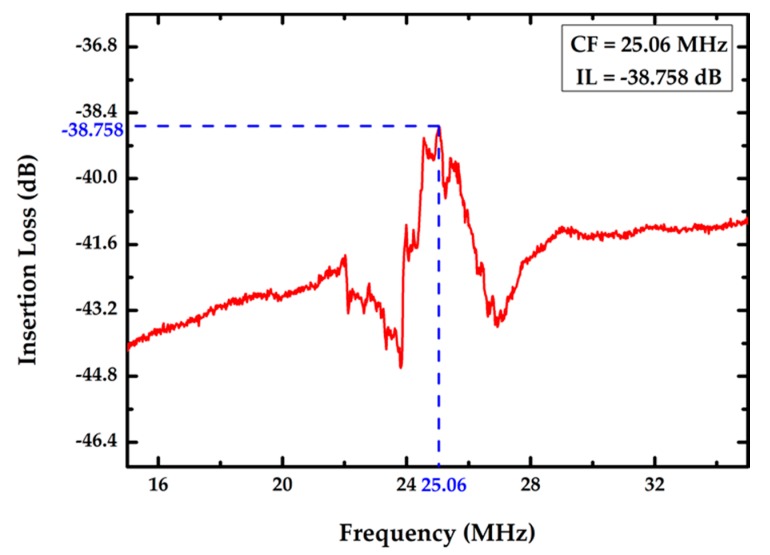
Frequency responses of the proposed FPW device.

A commercial Cascade RHM-06/V probe station and Agilent E5074 (Beaverton, OR, USA) network analyzer were used to measure the center frequency of the developed FPW device at room temperature. Two Cascade coplanar 150 ground–signal–ground (GSG) input probes were connected to the input and output IDTs of the FPW devices. As shown in Figure 5, the circular-type FPW devices have low insertion loss (−38.758 dB), low center frequency (25.06 MHz), and their signal-to-noise ratios are higher than those of conventional FPW devices.

**Figure 6:**
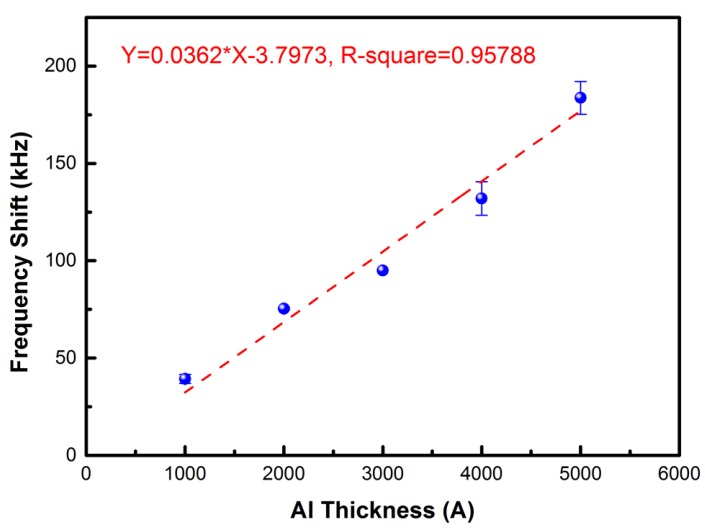
The frequency shifts of the FPW device with five different Al mass loadings.

The solid-state mass-sensitivity of the FPW devices was also investigated. Five different thicknesses of Al thin-film (from 1000 to 5000 Å and the thickness of pitch is 1000 Å) were deposited onto the surface of backside silicon to measure the changes of frequency. As shown in Figure 6, given the five different Al mass (from 27 to 135 μg/cm^2^ and each interval is 27 μg/cm^2^), the frequency shift of the circular FPW device is 39.33, 75.33, 95.0, 132.0, and 183.68 kHz, respectively. According to the reference [13], the mass loading of the floating thin plate, which causes change in resonant frequency—where $f_{0}$ denotes the center frequency of operation, Δf denotes the change of the resonant frequency due to a change in mass per unit area ($\Delta_{m}$), and Sm is the mass sensitivity of the FPW device—is given by the equation

$$\frac{\Delta f}{f_{0}}{\;=\;S}_{m}\Delta_{m}$$

The mass-sensitivity of the proposed FPW devices of circular IDTs/RGS are 126.67 cm^2^/g, and thus have the same high sensing linearity (R-square is 0.9579), which is beneficial to develop its associate readout circuit.

Table 2 is the comparison between the four sensing characteristics (detection time, limit of detection, linear range, testing equipment size) in the optimum compositions and those in previous studies [4,7,26]. Although the prior biosensing system has a lower limit of detection, the implemented FPW-based THC biosensing system presents the fastest response time, the widest linear range and the smallest testing system size. Therefore, the proposed biosensing system is more reliable, effective, portable, and suitable for early detection of THC.

### 3.2. Measurement of FPW Readout System Prototype

**Figure 7:**
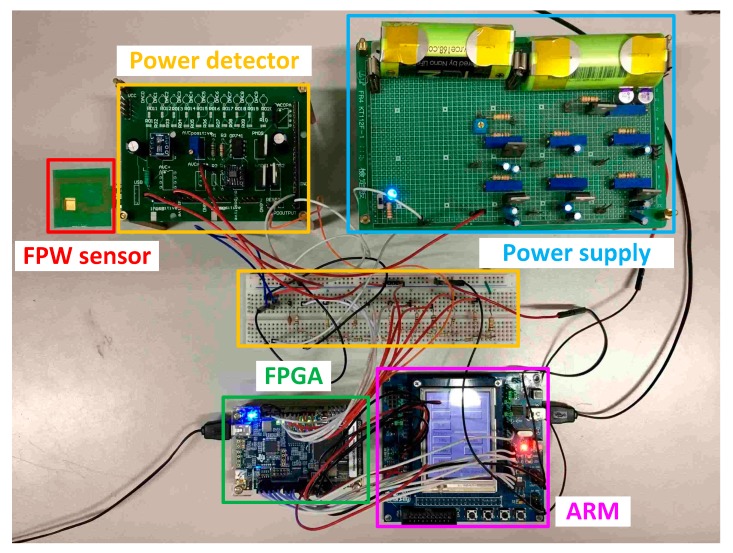
Photo of the system prototype measurement environment.

The FPW readout system prototype, as shown in Figure 7, is composed of a FPGA (control circuit and scanning signal generator), FPW sensor, power detector with discrete components (AVC, gain stage, and peak detector), and ARM board for displaying results. To increase the reliability, decoupling capacitors were coupled to restrain the noise; Li-ion batteries were also used in the power supply. 

**Figure 8:**
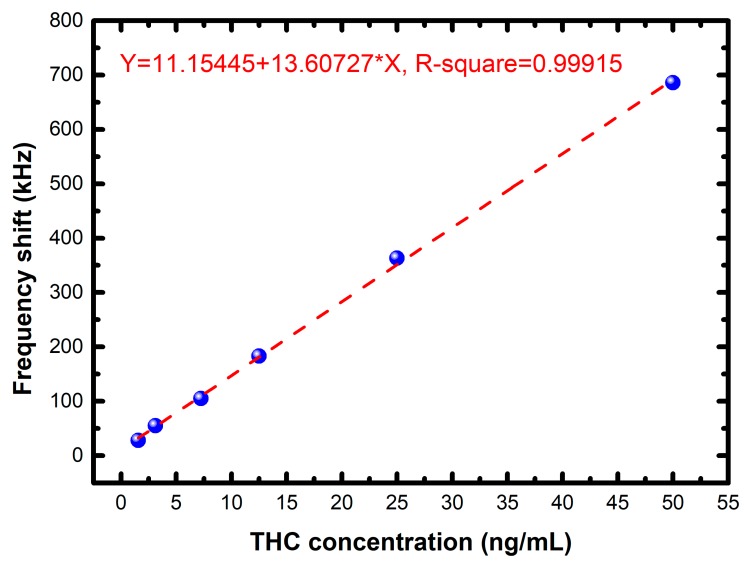
Measurement results of the FPW readout system prototype.

**Figure 9:**
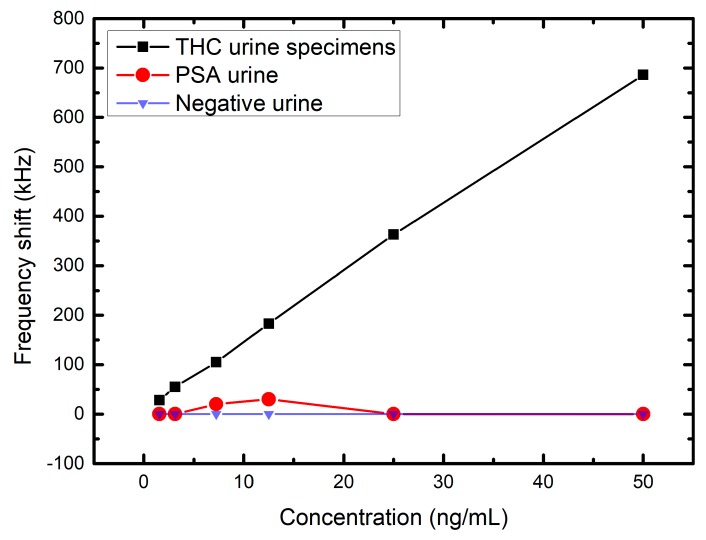
Measurement results of the THC urine specimens, PSA urine, and negative urine.

The output result of the FPW readout system prototype is shown in Figure 8 and Table 3, where six different concentration of THC urine specimens—e.g., 0, 1.5625, 3.125, 7.25, 12.5, 25, and 50 ng/mL—are measured. The y-axis is the measured average frequency shift (without the worst deviation data) of each concentration when the system is steady. The maximum error is 0.012 MHz and the linearity R-square is equal to 0.9992. The negative urine (without any protein or other biomolecules) is used as the negative control. As shown in Figure 9, the frequency shift is zero when the negative urine is measured. In additional, six different concentration of prostate specific antigen (PSA) urine are also measured in this paper, there are no frequency shift at 1.5625, 3.125, 25, and 50 ng/mL, and the frequency shifts are only 20–30 kHz at 7.25 and 12.5 ng/mL (which can be considered measurement errors). Notably, before every concentration is being tested, the FPW sensor will be rinsed first, and held until it dries out. Then the sensor is coated with THC antibody on the back-side silicon cavity again to ensure the precision of measurements.

The comparison between this work and the previous works [16,28] is shown in Table 4, where this work has the least error. Most important of all, only two OPAs (one in the gain stage and the other in the peak detector) are used, which effectively reduce the complexity in designing high bandwidth OPAs.

## 4. Conclusions

To reduce insertion loss of FPW devices, the circular-type IDTs/RGS configuration was proposed. Thanks to the low insertion loss (−38.758 dB) and low center frequency (25.06 MHz) of this novel device, development for a readout IC and bio-sensing microsystem can be easier. Additionally, a novel FPW-based THC biosensor was developed using MEMS and SAMs technologies. The realized FPW-THC biosensors have a low detection limit for THC antigen (1.5625 ng/mL), a short response time (<10 min), and a high mass-sensitivity (126.67 cm^2^/g for circular IDTs/RGS). Moreover, based on the developed FPW devices, the FPW readout system for THC detection was prototyped, and has been proven to attain higher linearity (R-square = 0.9992) and less OPAs (only 2). Thanks to the proposed AVC circuit, the coupling problems of AC and DC components were also resolved.

## Figures and Tables

**Table 1:** Comparison of four main types of acoustic sensors.

Device	Mass Sensitivity (cm^2^/g)	Motion at Surface	Wave Velocity (Relative to Liquid)	Operating Frequency (MHz)
TSM	1–10	Transverse	Fast	1–10
SAW	100–200	Transverse and normal	Fast	30–300
APM	20–40	Transverse	Fast	25–200
FPW	100–1000	Transverse and normal	Slow	2–20

**Table 2:** Comparison of this research with the previous works of THC biosensor.

References	This Work	[26]	[7]	[27]
Technology	FPW	ELISA	HPLC	GC/MS
Year	2017	2017	2015	2014
Detection time	<10 min	<2 h	>20 min	>1 h
Limit of detection	1.5625 ng/mL	0.1 ng/mL	10 ng/mL	0.1 ng/mg
Linear range	1.5625–50 ng/mL	0.05–100 ng/mL	10–104 ng/mL	0.16–2.3 ng/mg
Testing equipment size	Portable	Non-portable	Massive equipment	Massive equipment

**Table 3:** The frequency shifts of the FPW-based THC biosensors are measured under six concentrations.

THC Concentration (ng/mL)	Frequency Shift (kHz)
1.5625	28
3.125	55
7.25	105
12.5	183
25	363
50	686

**Table 4:** Comparison with the previous works.

References	[16]	[28]	This Work
Year	2013	2014	2017
Technology	FPGA & chip	FPGA & chip	FPGA & discrete components
Linearity	N/A	0.9772	0.9992
Maximum error	N/A	0.12 MHz	0.012 MHz
Number of OPA	4 (at least)	4 (at least)	2
